# The Anti-Scar Effects of Basic Fibroblast Growth Factor on the Wound Repair *In Vitro* and *In Vivo*

**DOI:** 10.1371/journal.pone.0059966

**Published:** 2013-04-02

**Authors:** Hong-Xue Shi, Cai Lin, Bei-Bei Lin, Zhou-Guang Wang, Hong-Yu Zhang, Fen-Zan Wu, Yi Cheng, Li-Jun Xiang, Di-Jiong Guo, Xu Luo, Guo-You Zhang, Xiao-Bing Fu, Saverio Bellusci, Xiao-Kun Li, Jian Xiao

**Affiliations:** 1 School of Pharmacy, Key Laboratory of Biotechnology and Pharmaceutical Engineering, Wenzhou Medical College, Wenzhou, P. R. China; 2 Department of Burns, The First Affiliated Hospital, Wenzhou Medical College, Wenzhou, P. R. China; 3 Department of General Surgery, Cixi Hospital, Wenzhou Medical College, Ningbo, P. R. China; 4 Department of Orthopaedics, The Second Affiliated Hospital, Wenzhou Medical College, Wenzhou, P. R. China; 5 Wound Healing and Cell Biology Laboratory, Institute of Basic Medical Science, Chinese PLA General Hospital, Beijing, P. R. China; 6 German Center for Lung Research, Universities of Giessen, Giessen, Hessen, Germany; University of Hong Kong, China

## Abstract

Hypertrophic scars (HTS) and keloids are challenging problems. Their pathogenesis results from an overproduction of fibroblasts and excessive deposition of collagen. Studies suggest a possible anti-scarring effect of basic fibroblast growth factor (bFGF) during wound healing, but the precise mechanisms of bFGF are still unclear. In view of this, we investigated the therapeutic effects of bFGF on HTS animal model as well as human scar fibroblasts (HSF) model. We show that bFGF promoted wound healing and reduced the area of flattened non-pathological scars in rat skin wounds and HTS in the rabbit ear. We provide evidence of a new therapeutic strategy: bFGF administration for the treatment of HTS. The scar elevation index (SEI) and epidermal thickness index (ETI) was also significantly reduced. Histological reveal that bFGF exhibited significant amelioration of the collagen tissue. bFGF regulated extracellular matrix (ECM) synthesis and degradation via interference in the collagen distribution, the α-smooth muscle actin (α-SMA) and transforming growth factor-1 (TGF-β1) expression. In addition, bFGF reduced scarring and promoted wound healing by inhibiting TGFβ1/SMAD-dependent pathway. The levels of fibronectin (FN), tissue inhibitor of metalloproteinase-1 (TIMP-1) collagen I, and collagen III were evidently decreased, and matrix metalloproteinase-1 (MMP-1) and apoptosis cells were markedly increased. These results suggest that bFGF possesses favorable therapeutic effects on hypertrophic scars *in vitro* and *in vivo*, which may be an effective cure for human hypertrophic scars.

## Introduction

During the repair of a wound proceeds, keloid and hypertrophic scars (HTS) are a common problem. Clinically, they are characterized by excessive deposition of collagen in the dermis and subcutaneous tissues secondary to traumatic. This process is regulated by cytokines and growth factors such as transforming growth factor β (TGF-β), epidermal growth factor (EGF), fibroblast growth factor (FGF) and platelet-derived growth factor (PDGF) [1]. During embryo-genesis, FGFs play key roles in regulating cell proliferation, migration, and differentiation. In adult tissues, FGFs have various effects, including mediating angiogenesis and neuroprotection, in addition to their stimulatory effects during wound repair [2], [3], [4]. Basic fibroblast growth factor (bFGF) is a potent mitogen and chemoattractant for endothelial cells, fibroblasts and keratinocyte. bFGF stimulates the metabolism, growth of the extracellular matrix (ECM), and the movement of mesodermally derived cells [5]. The administration of recombinant bFGF to skin wounds can accelerate acute and chronic wound healing [6], [7], [8], [9]. In addition, bFGF-knockout mice delayed healing of skin wounds [10], which means that bFGF plays a key role for wound healing.

The goal for wound treatment is the fast and scarless healing, although this is quite difficult for adult tissues. The accelerating wound healing may improve the quality of healing and alleviate the scar. The anti-scarring effects of bFGF have been shown in both animal models and clinical use; postoperative administration of bFGF also inhibits hyperplastic scar without side effects [6], [11], [12], [13]. These results suggest that bFGF treatment during wound healing may exert anti-fibrosis effects in keloids. However, little is known about the precise pathological mechanisms of bFGF on the prevention of HTS and keloid formation; thus, the underlying mechanism deserves further investigations.

During the wound healing process, the imbalance of collagen synthesis and degradation resulting in excess accumulation of dermal collagen can lead to the scar complications [14]. Sufficient content of type III collagen may prevent scar tissue formation, while excessive secretion of type I collagen may result in a disorganized fiber structure and hypertrophic scar formation [15]. In addition, α-smooth muscle actin (α-SMA) also plays a major role in the fibrosis, as sustained the myofibroblasts form the granulation tissue [16]. Additionally, TGF-β is particularly important for the fibrosis. Localized increase in the release and activation of TGF-β1 in burn injuries have delayed reepithelialization and enhanced the scarring response [17], TGF-β1 and TGF-β2 induce cuaneous scarring, whereas TGF-β3 seems to inhibit this effect [18], [19]. Therefore, collagen distribution, α-SMA expression, and the TGF-β1 mediated signal pathway are analyzed as the mechanisms of anti-scarring effect of bFGF.

This study aims to demonstrate whether bFGF can alleviate or eliminate formed hypertrophic scars in a full-thickness excisional rat model and in the rabbit ear model. To explore the possible mechanism of action, we comparatively evaluated the *in vitro* effects of bFGF on fibroblasts isolated from human HTS and normal skin. Thus, we provide evidence of a new therapeutic strategy: bFGF administration for the treatment of established HTS.

## Results

### bFGF Accelerates Acute Wound Closure in the Rat Incised Injury Model

**Figure 1:**
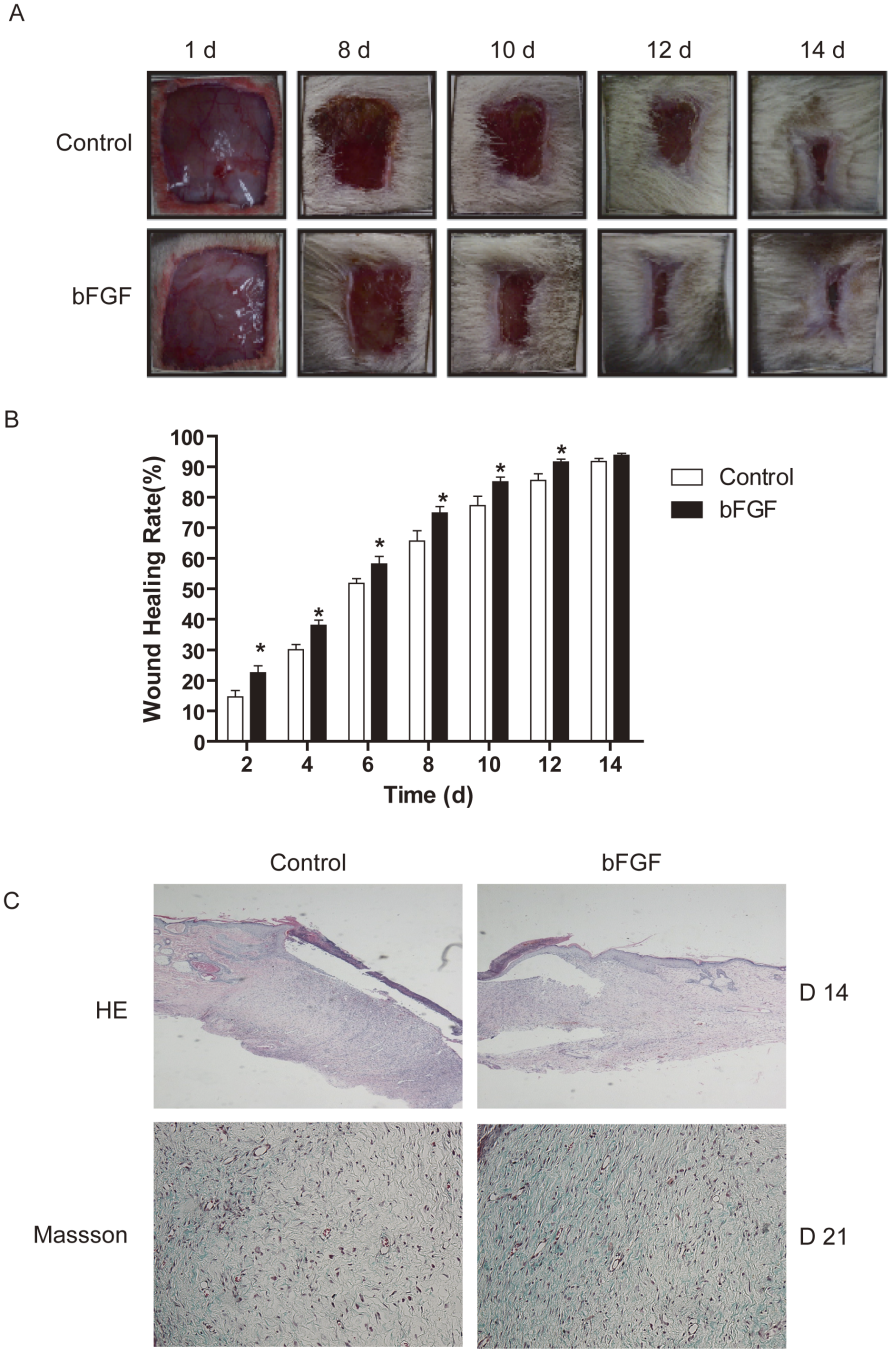
Wound closure and histopathological characteristics of bFGF treated wound healing in rat. (A) Representative photographs of full-thickness skin wounds at various time points after treatment with or without 1 µg/ml bFGF. (B) The wound healing rates of bFGF. *P<0.05 compared to control group, n = 8. (C) Histopathological observation and masson staining of collagen in wound healing at day 14 post-wounding (×200).

Wound healing of the skin incision was determined by the percentage of wound surface covered by regenerating epidermis. The wounds treated by bFGF recovered much more quickly with better skin appearance (Figure 1A). After day 8, the wounds treated with bFGF were almost scarless, while the wounds in the control had obvious scars. Thus, bFGF significantly contributed to wound healing, compared to the control group (*P*<0.05; Figure 1B). After day 14, both the bFGF group and the control group showed wound closure mostly, for natural repair of the rat skin. These wound closure rates made a good match to the results of HE staining and Masson Staining (Figure 1C). Longer keratinocyte migration tongue and collagen expression was observed on day 14 for bFGF-treated wounds; the results demonstrated the accelerating effect of bFGF on the wound re-epithelization *in vivo*.

**Figure 2:**
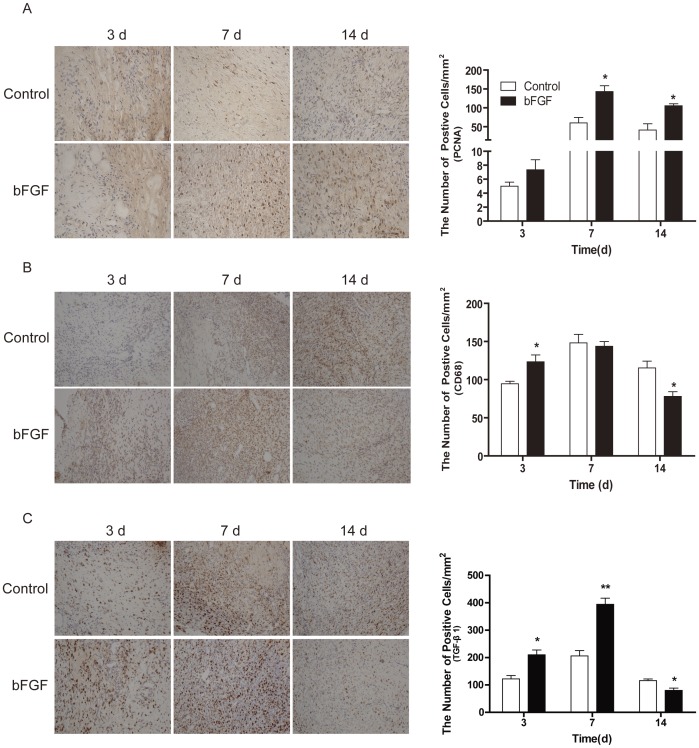
The expression of PCNA, CD68 and TGF-β1 after bFGF treatment. Immunohistochemistry of (A) PCNA, (B) CD68 and (C) TGF-β1 was performed on the indicated day (×200); the histogram represents the positive cells and optical density of the immunohistochemistry results. *P<0.05 and **P<0.01 compared to control group, n = 8.

Compared with the control group, expression amounts of PCNA and TGF-β1 cell proliferation proteins in the bFGF group significantly increased after establishment of the ulcer model on day 7. On the 14^th^ day, the expression amounts of PCNA were remain increase in bFGF group, while the TGF-β1 levels were decreased in the bFGF group (Figure 2A and 2C). Inflammatory response is instrumental to supplying growth factor and cytokine signals that orchestrate the cell and tissue movements necessary for repair during wound healing, As a macrophage marker, CD68 belongs to a family of acidic, high glucosylated lysosomal glycoproteins (GLPs). Immunohistochemical staining of CD68 indicated upregulated inflammatory response on day 3 and 7 and downregulated the expression on day 14 in the bFGF group (Figure 2B). These results suggest that bFGF improved wound healing by stimulating fibroblast growth, reducing scar formation, and regulating the inflammation response.

### bFGF Alleviates the Scar Formation in the Rabbit Ear Model

**Figure 3:**
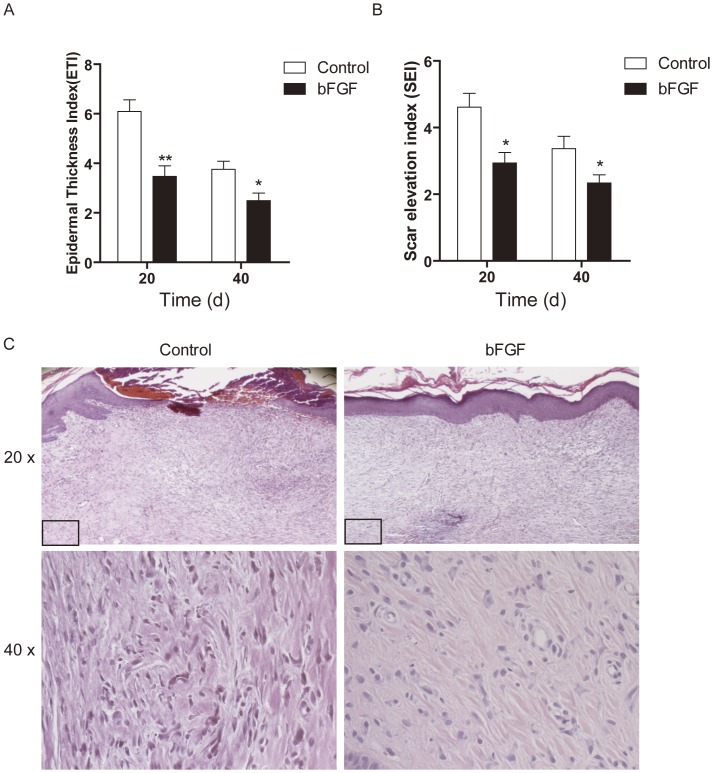
bFGF alleviated the scar formation in rabbit ear model. (A) The averaged Epidermal Thickness Index (ETI) of the scars. Epidermal hypertrophy was displayed by ETI. ETI >1 depicts a hypertrophic epidermis. (B) The averaged scar elevation index (SEI) of the scars. Dermal hypertrophy is displayed by the SEI, where SEI >1 depicts a hypertrophic scar. *P<0.05, **P<0.01 compared to control group, n = 6. (C) The microscopic histology of wounds that control or bFGF at day 40, HE stain.

All wounds had adequate scar maturation and showed histologic evidence of scarring. The mean scar thickness (SEI) in the control group was 4.612±0.4152 and 3.369±0.3712, which was higher than that of the bFGF group with 2.939±0.3131, 2.338±0.2446 on days 20 and 40, respectively (Figure 3A). The mean epidermal thickness (ETI) of the control group was 6.089±0.4744 and 3.758±0.3262 compared with 3.472±0.4232 and 2.490±0.3070 for the bFGF group (*P*<0.05; Figure 3B). This represents a significant epidermal thickness reduction in wounds treated with bFGF.

Histologically, the dermis layer of the control scars thickened significantly, and the boundary between the papillary and reticular layers of dermis was obscure; collagen fibers were dense, with derangements in collagen bundles, which were irregularly arranged in the profound dermis and nodular, circular, or whirled in the superficial dermis. The number of cells also increased, while the basal layer of the epidermis in the scars treated with bFGF for 40 day was flattened. The dermis layer was not significantly thickened, and collagen fibers were well arranged, with few cells (Figure 3C).

### Effect of bFGF on Collagen I and Collagen III Synthesis

**Figure 4:**
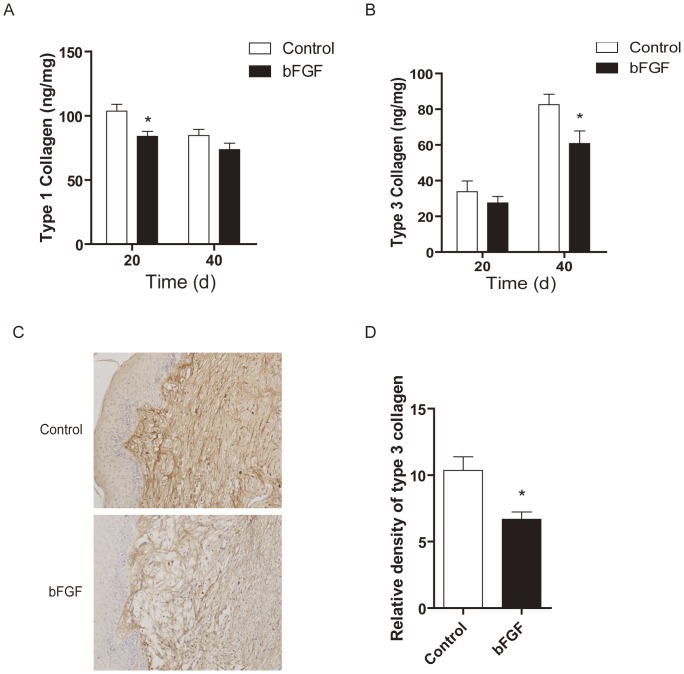
bFGF decreased collagen I and collagen III synthesis. The levels of expression of (A) collagen Ι and (B) collagen III in scars treated with saline or bFGF, *P<0.05, **P<0.01 compared to control group, n = 6. (C) Immunohistochemistry of the expression of collagen III in scars treated with saline or bFGF (×200). (D) Analysis of relative density collagen III, *P<0.05, **P<0.01 compared to control group, n = 6.

In order to evaluate the molecular effects of bFGF on matrix production, we measured protein expression of collagen Ι and III, which constitute the bulk of the scar ECM. As expected, collagen I expression was significantly decreased on day 20 (*P*<0.05), while collagen I expression was insignificantly decreased on day 40 (Figure 4A); collagen III significantly reduced on day 40 (*P*<0.05) in the bFGF treated group (Figure 4B). The collagen density was significantly reduced in wounds treated with bFGF compared with the untreated wounds by immunohistochemical staining of the collagen III on day 40 (*P*<0.05; Figure 4C and 4D).

### Effect of bFGF on on α-SMA and TGF-β1 Expression

**Figure 5:**
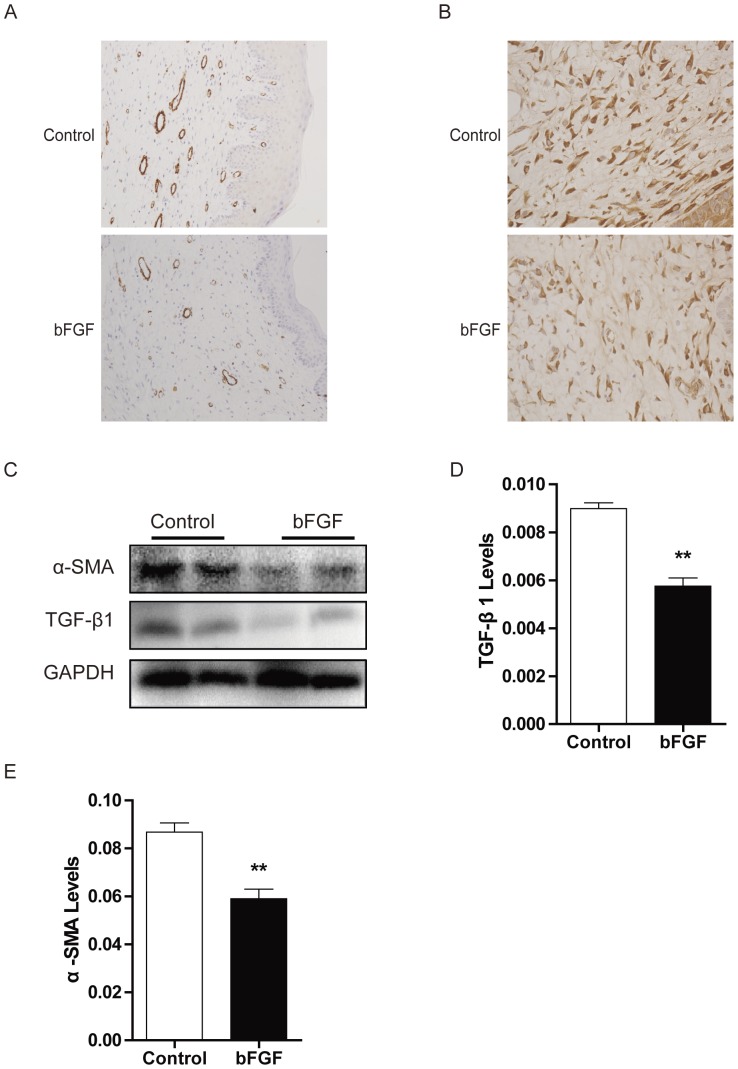
bFGF decreased α-SMA and TGF-β1 expression in scars. Immunohistochemistry of the expression of (A) α-SMA and (B) TGF-β1 in scars treated with saline or bFGF in scars. (C) The levels of α-SMA and TGF-β1 in scars treated with saline or bFGF by Western blot. Analysis of the relative protein of (E) α-SMA and (F) TGF-β1 was performed. **P<0.01 compared to control group.

The persistent presence of myofibroblasts is a distinctive feature of HTS that contributes to the excessive matrix production. In untreated scars, immunohistochemistry revealed significantly staining for α-SMA. The positive number of α-SMA in the scars treated with bFGF was significant decreased compared with the control group on day 40 (Figure 5A). Western blot analysis showed that levels of α-SMA expression decreased in the bFGF groups relative to the control group (Figure 5C and 5E). The expression of TGF-β1 in the scars treated with bFGF decreased compared with the control group after immunohistochemical staining of the TGF-β1 on day 40 (Figure 5B). Western blot analysis showed that the level of TGF-β1 expression significantly decreased in the bFGF group compared with the control groups on day 40 (Figure 5C and 5D).

### Effects of bFGF on mRNA Expression of HSF

**Figure 6:**
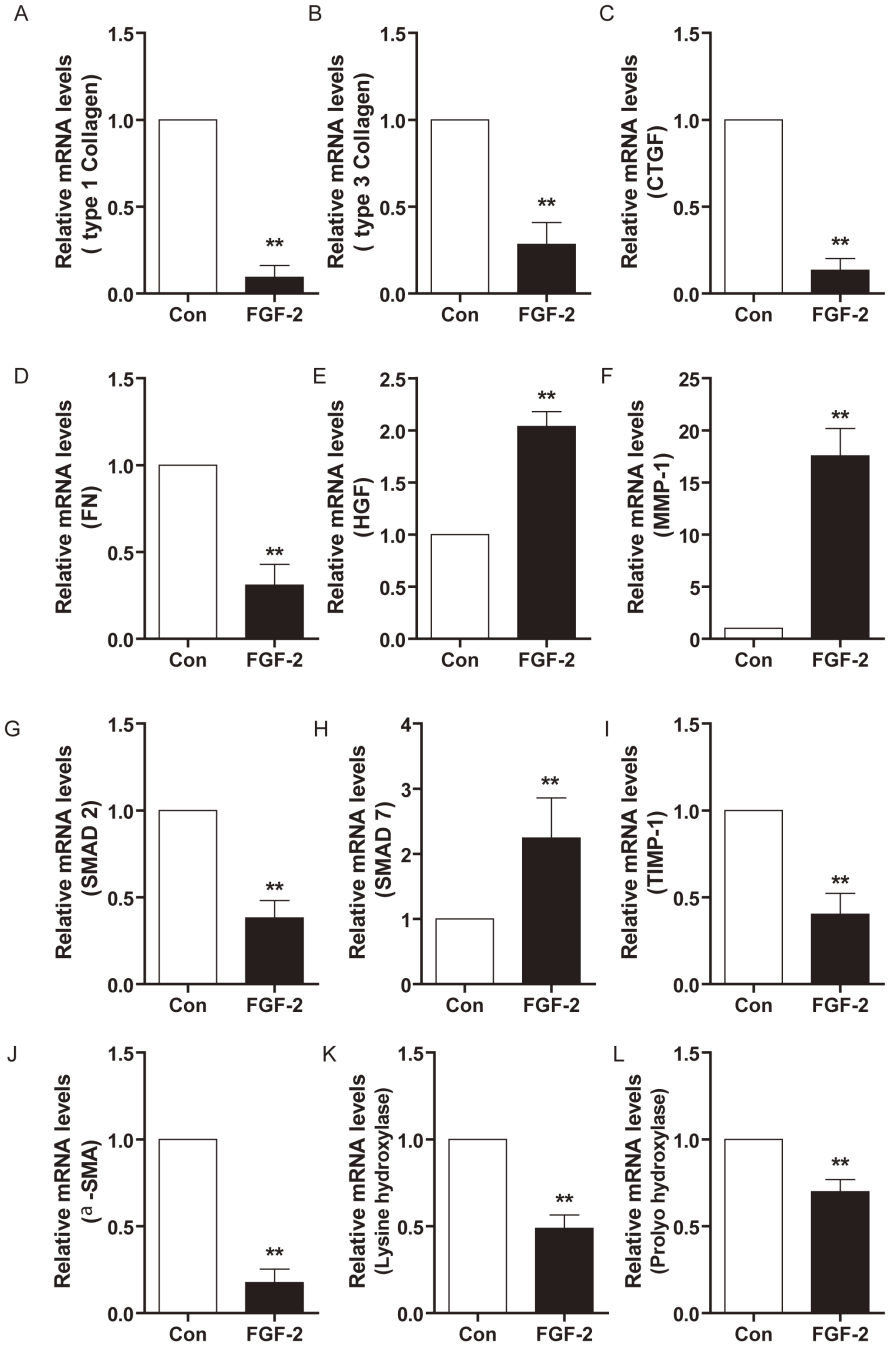
Effects of bFGF on mRNA levels in HSF. RT-PCR analysis the mRNA levels of type collagen Ι and (B) collagen III, (C) CTGF, (D) HGF, (E) FN, (F) MMP-1, (G) TIMP-1, (H) SMAD-2, (I) SMAD-7, (J) α-SMA, (K) Lysine hydroxylases and (L) Prolyo hydroxylase in HSF treated with bFGF or saline for 5 days. *P<0.05 compared to control group.

bFGF stimulated cell proliferation of both HTS-derived and normal skin-derived fibroblasts (Data not shown). Real-time PCR indicated that bFGF downregulated FN, collagen I and III, α-SMA, and hydroxylases expression in HTS-derived fibroblasts (Figure 6A, 6B, 6E, 6J, 6K and 6L). The MMP-1 mRNA expression increased in scar fibroblasts treated with bFGF. As the natural inhibitor of the MMPs, TIMP-1 was downregulated in the bFGF group (Figure 6E and 6F). Most interestingly, bFGF highly upregulated gene expression of HGF, but downregulated CTGF levels (Figure 6C and 6D).

### bFGF Induces Cells Apoptosis and Inhibits TGF-β1 Mediated SMAD Signaling

**Figure 7:**
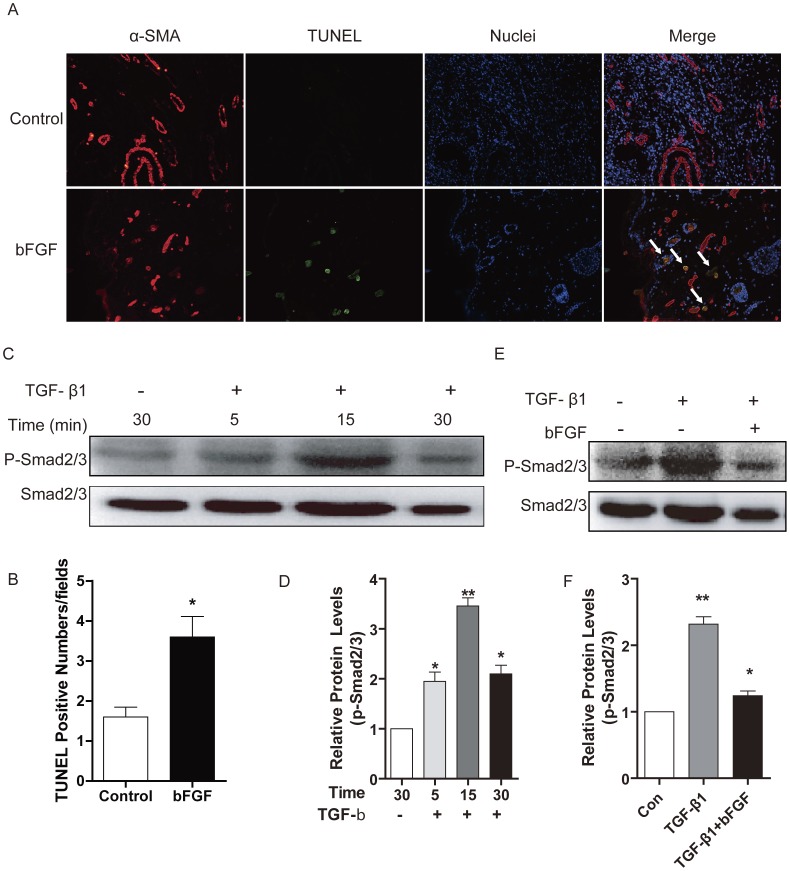
bFGF induced cells apoptosis and inhibited TGF-β1/SMAD signaling pathway. (A and B) TUNEL staining analysis of the apoptosis cells in rat skin by bFGF or saline in day 14 after wounding. The nuclear was labeled by Hoechst (blue), the myofibroblast was labeled by α-SMA (red), the apoptosis cells was labeled by TUNEL (green). Colocalization of α-SMA and TUNEL indicate apoptosis myofibroblast (white arrow) (×200). *P<0.05 compared to control. (C and D) Western blot analysis of the phosphorylation of SMAD2/3 (Ser 423/425) in HSF incubated with TGF-β1 (5 ng/ml). (E and F) Western blot analysis of the phosphorylation of SMAD2/3 in HSF incubated with TGF-β1 and bFGF (10 ng/ml). *P<0.05, **P<0.01 compared to control group.

To determine the level of apoptosis in wounds filled with collagen, TUNEL staining was performed. Comparing the TUNEL positive numbers of the bFGF group with those of the control group on day 14, bFGF significantly increased the TUNEL positive numbers (Figure 7A and 7B). The results suggest that bFGF treatment is a potent stimulator of myofibroblast apoptosis.

SMAD proteins as intracellular effectors of TGF-β1 signaling, SMAD7 as the unique negative feedback regulator of TGF-β1 signal pathway prevents SMAD2/3-receptor interactions and subsequent SMAD phosphorylation, and degrade receptor complexes. In this study, gene expression of SMAD7 was markedly upregulated by bFGF in HSF, while the gene expression of SMAD2 was significantly downregulated (Figure 6H and 6I). Western blot analysis showed that the phosphorylation of SMAD2/3 was upregulated in fibroblasts treated with TGF-β1 (Figure 7C and 7D), while fibroblasts incubated with TGF-β1 and bFGF showed a significant decrease of phosphorylation of SMAD2/3 (Fig 7E and 7F), which means that bFGF inhibited the TGF-β/SMAD signal pathway in HTS.

## Discussion

Scarring is a multifactorial process with different clinical presentations that affects more than 40 million people worldwide [20]. Generally, scars can be classified into two categories, pathological scars and non-pathological scars. There are some preventive and therapeutic measures for exuberant scars, such as silicone, pressure therapy, corticosteroids, laser therapy, cryotherapy, radiation, and surgery [21], [22], [23], [24]. However, there is no consensus about the best treatment for complete and permanent improvement of scars with few side effects.

Funato et al. [25] reported that bFGF is a possible inducer of apoptosis in myofibroblasts during palatal scar formation. Spyrou and Naylor [26] found that treatment with bFGF inhibited the transient phenotypic change of granulation-tissue fibroblasts into myofibroblasts. In order to identify a means to reduce scar formation of the skin after incision, Ono et al. [11] examined the effect of local administration of bFGF in humans. They found that no patient treated with bFGF had hypertrophic scars, while some patients had hypertrophic or very wide scars in the control group; the ratios of minimum scarring for the bFGF treatment group were statistically significantly higher than those of control group. In this study, we evalutate the therapeutic potential of bFGF for wound healing and HTS animal model as well as human scar fibroblast cell model and analyze the potential mechanisms. We find that bFGF promoted wound healing and reduced the area of flattened non-pathological scars in rat skin wounds (Figure 1) as well as the size of hypertrophic scars in the rabbit ear (Figure 3). In the early stages of incisional-wound healing, bFGF administration has no adverse effect on the tensile strength of the wound and, at later stages, can be attributed to the improved the architecture of the neodermis.

Type I and III collagens are the central components of ECM products. However, the production of collagen can be a double-edged sword: on the one hand, it is necessary for wound healing; on the other hand, excess deposition of collagen can result in scarring [27], [28]. Therefore, the appropriate expression of collagen is required for ideal wound healing. Our findings indicate that bFGF can accelerate wound healing with increasing collagen production and subsequent collagen deposition (Figure 1). The outcome is an improvement in the quality of wound healing. In the rabbit hypertrophic scar model, bFGF resulted in a sparse arrangement of the collagen, similar to normal skin collagen distribution, and reduced type I collagen and type III collagen content, which prevented the formation of nodular structures (Figure 4). In the HSF, bFGF reduced collagen gene expression (Figure 6A and 6B). Lysyl hydroxylase and prolyl hydroxylase, as the key enzymes for the formation and stabilization of collagen, also downregulated in bFGF treatment (Figure 6K and 6L). These results support the alternative effect of bFGF on collagen expression in the different stages of wound healing, which is beneficial for wound closure and scar diminution. Also, the study by Xie et al. [11] supports the potential of bFGF to accelerate wound healing and improve the quality of scars by regulating the balance of collagen synthesis and degradation.

Consequently, interfering with one or several components of the ECM metabolism could be a potential therapeutic intervention to alleviate scars [1], [14]. The amount of ECM in the tissue might be controlled through a balance among ECM production, ECM degradation by MMPs, and inhibition of MMPs by tissue inhibitors of metalloproteinases [14], [29]. Our study shows that the stimulation of HSF with bFGF *in vitro* could result in an upregulation of MMP-1 and decreased TIMP-1 (Figure 6F and 6G). Fibronectin is one of the most important ingredients of the ECM and plays a particularly important role in wound repair, largely determining the quality of the wound [30], [31]. The deposition and/or polymerization of fibronectin into the ECM controls the deposition and stability of other ECM molecules [32], [33]. Our data show that bFGF decreased FN gene expression in HSF (Figure 6E). All of these results indicate that bFGF regulates ECM metabolism to improve wound healing and hypertrophic scarring.

Myofibroblasts, which elaborate matrix proteins and initiate wound contraction, are transiently present in healing wounds. The persistent presence of myofibroblasts is a distinctive feature of HTS and contributes to excessive matrix production [21], [34]. Differentiation of fibroblasts into myofibroblasts is closely associated with α-SMA [17], [35]. Our results indicate down-regulation of α-SMA expression by bFGF in the HSF and rabbit model (Figure 5 and 6J). It has been demonstrated that TGF-β1 promoted α-SMA expression [36], [37]. The present study also investigated the degree of apoptosis in fibroblastic cells and levels of TGF-β1 expression in scars. Immunofluorescence and western blot show that inclusion of bFGF significantly increased apoptosis in granulation tissue cells of the wound, consistent with other reports [12]. The SMAD2/SMAD3 signaling system of the TGFβ1/SMAD-dependent pathway is considered an important pathway in scar formation. bFGF treatment also markedly decreased SMAD2/3 phosphorylation in the HSF (Figure 7). This result indicates that bFGF treatment can increase apoptosis of myofibroblasts, leading to inhibition of scar formation due to inhibition of the TGF-β1 signaling pathway. Taken together, these findings suggest that bFGF reduce scarring and promote wound healing by inhibiting the TGFβ1/SMAD-dependent pathway. Thus bFGF might be applicable as an anti-scarring agent after the surgery of skin or other organs where myofibroblast overgrowth would induce complications for scarring.

In conclusion, we provide evidence of a new therapeutic strategy, bFGF administration for the treatment of HTS. bFGF regulate ECM synthesis and degradation via interference in the MMP-1, TIMP-1, lysyl hydroxylase and prolyl hydroxylase gene expression. The efficacy of treatment using bFGF was also demonstrated in animal models and the human cell model. However, it no doubt that the limitations of bFGF in scar therapy still need further investigation and improvement. For example, single a dose of bFGF was treated right after injury, a post-injury treatment of optimal dose and extended time would better evaluate the therapeutic value in the future. It is interesting that although we have tried several concentrations of bFGF in our model *in vivo*, there is no obvious enhancement of the protective effect with the increase of bFGF, which maybe related to the regulation of absorption and metabolism. Nevertheless, the anti-scarring effect of bFGF in the therapy of HTS is confirmative and feasible, to improve the pharmacodynamic action and demonstrate the mechanism underlying is necessary in the following study.

## Materials and Methods

### Ethics Statement

All animals were from the Laboratory Animals Center of Wenzhou Medical College, and treated strictly in accordance with international ethical guidelines and the National Institutes of Health Guide concerning the Care and Use of Laboratory Animals. The experiments were carried out with the approval of the Animal Experimentation Ethics Committee of Wenzhou Medical College.

Hypertrophic scar patients were selected according to the Vancouver Scar Scale (VSS) ranging from 10 to 13. All patients were informed of the purpose and procedure of this study and agreed to offer their tissue specimens. Written consent was obtained from all participants involved in this study. All protocols were approved by the Ethics Committee of First Affiliated Hospital of Wenzhou Medical College, Wenzhou, China.

### Animal Model

Male Sprague-Dawley rats (n = 30), weighing 250 g, were chosen for the experiment. The skin was cleaned with alcohol and two fullthickness wounds (2 cm×2 cm) extending through the panniculus carnosus were made on the dorsum on each side of midline under aseptic conditions. bFGF was obtained from the Key Laboratory of Biotechnology and Pharmaceutical Engineering in Wenzhou Medical College and applied to the wounds of the experimental group (1 ml for each wound) every other day in concentrations of 1 µg/ml (dissolved in 0.9% w/v saline), while the control group received equal amounts of 0.9% w/v saline treatment for 14 days. Wounds were left uncovered after injury, and wound areas were measured at various time points [9]. The rate of wound closure was calculated using the following formula: Wound closure rate = [(Original wound area-Open area on final day)/Original wound area×100%.

The rabbit ear model of hypertrophic scarring was established as described previously with a minor modification [23]. In brief, 12 Japanese white rabbits (no sex restriction), weighing 2.5 to 3.0 kg, were kept under standard conditions, anesthetized with xylazine (5 mg/kg) and prepared for wounding under sterile conditions. Five 1 cm fullthickness circular wounds were created down to the cartilage on the ventral side of each ear using a 1 cm punch biopsy. The perichondrial membrane was then dissected off the cartilage using a surgical blade. The bFGF solution was applied to the wounds of the experimental group (50 µL to each wound) every day in concentrations of 1.2 µg/ml (dissolved in 0.9% w/v saline) for 7 days, while the control group received equal amounts of 0.9% w/v saline.

### Histological Examination and Immunohistochemistry Staining

Histological analysis of the skin was performed as previously described [9]. The scar or skin tissues were fixed in 4% paraformaldehyde at 4°C overnight prior to processing for paraffin embedding, cut in 5 µm sections, and stained with hematoxylin-eosin (HE) or Masson’s Trichrome Stain Kit (Sigma–Aldrich, St. Louis, MO). The other half was stored at –80°C for protein extraction. The scar elevation index (SEI) and epidermal thickness index (ETI) were used for histomorphometric analysis and measured for treated and untreated wounds. The SEI is a ratio of total wound area tissue height to the area of normal tissue below the scar. The height of the normal tissue is determined based on the height of the adjacent unwounded dermis. All measurements were taken within the confines of the wounded area under 40× magnification from the HE stained tissue sections. An SEI of 1 indicates that no newly hypertrophied dermis formed, whereas an index >1 indicates HTS formation. The ETI was used to determine the degree of epidermal hypertrophy and was based on measurements taken from H&E-stained tissue sections at 400× magnification. The ETI is a ratio of averaged epidermal height in scar tissue to the averaged epidermal height in normal uninjured skin. ETI >1 indicates hypertrophic epidermis formation.

The immunohistochemical staining of the PCNA, CD68, TGF-β1, α-SMA and collagen III (Santa Cruz Biotech, Santa Cruz, CA) was conducted by respective antibody. Sections were dewaxed and hydrated; endogenous peroxidase was blocked with 3% hydrogen peroxide for 10 min; nonspecific binding was blocked with 1% BSA for 30 min. Primaries were applied for PCNA (1∶100), CD68 (1∶100), TGF-β1 (1∶150), α-SMA (1∶50), and collagen III (1∶200) overnight at 4°C. Biotinylated secondary antibodies were then applied at 1∶200 for 30 min, followed by incubation with horseradish peroxidase (HRP)-streptavidin at 1∶400 for 30 min. Color development was performed with DAB for 3 to 5 min for all samples, followed by haematoxylin counterstaining, dehydration and coverslipping. The immunopositive in fields was counted for per sections using Image-Pro Plus software (Nikon, Tokyo, Japan).

### Terminal Deoxynucleotidyl Transferase (TdT)-mediated dUTP Nick-end Labeling (TUNEL) Staining

TUNEL was performed using a commercial kit (Roche, Mannheim, Germany) according to the manufacturer's instructions. Negative controls had the TdT substrate omitted from the buffer solution. Following staining by the TUNEL protocol, sections were incubated overnight at 4°C with rabbit anti-α-SMA at a dilution of 1∶ 200; a secondary antibody were then applied at 1∶400 for 60 min at 37°C. Fluorescence was examined using a Nikon microscope (Nikon, Tokyo, Japan) equipped with a reflected light fluorescence device. Negative control experiments, in which anti-α-SMA was replaced by rabbit immunoglobulin at the same final concentration, consistently showed no staining.

### Collagen I and Collagen III Quantification

The quantification of collagen Ι and III used an ELISA Kit (R & D Systems Inc., Minneapolis, MN, USA) according to the operation manual. In brief, 100 µL of standards or samples were added to the appropriate well of the antibody pre-coated microtiter plate, followed by 50 µL of conjugate; each well was then covered and incubated for 1 h at 37°C. Next, 50 µL of substrates A and B were added to each well and incubated for 10 min at room temperature (to avoid sunlight). Finally, added 50 µL of the stop solution was added, and the optical density (O.D.) was read at 450 nm using a microtiter plate reader immediately.

### Western Blot

Western blotting was performed as previously described [38]. Briefly, the scar tissues or the cells were lysed; then the protein samples were denatured and then separated on 10.6% polyacrylamide gels, and transferred to the polyvinylidene difluoride membrane. The membranes were incubated in TBS containing 5% nonfat milk and 0.05% Tween-20 for 1 h at room temperature and blotted with primary antibodies (dilution 1∶300) at 4°C overnight. The membranes were washed with TBST for 15 min the next day. Subsequently, the membranes were incubated with horseradish peroxidase-conjugated affinipure goat anti-rabbit antibody (dilution 1∶3000) for 1 h at room temperature and washed with TBST for 21 min. The membranes were then detected using ECL. The western blot results were further analyzed with Quantity One software 4.1.1 (Bio-Rad, Hercules, CA).

### Fibroblasts Culture

Primary culture of hypertrophic scar fibroblasts (HSF) was established from the patients who had received no previous treatment for burn hypertrophic scar before surgical excision as described [17]. In briefly, the specimens were washed three times in a phosphate-buffered saline (PBS) solution containing 1% penicillin, streptomycin sulfate. Subsequently, the tissues were digested in 0.5% Dispase II overnight at 4°C. The epidermis and subcutaneous tissue were excised from the tissues, which were cut into approximately 1× 1× 0.5 cm and placed as explants in T25 tissue culture flasks. Dulbecco’s modified eagle medium (DMEM) containing 1.0 g/L D-glucose, 10% fetal bovine serum (FBS), 1% penicillin, streptomycin sulfate and 2 mM L-glutamine was used as the growth medium. The medium was replaced every 3 days. The primary fibroblasts were grown at 37°C in an atmosphere of 5% CO_2_ and were passaged every 2 days by trypsinization. Cells were used for experiments at passages three to six.

### Quantitative Real-Time PCR (RT-PCR)

After HSF was treated with bFGF or saline for 5 days, total RNA from HSF was prepared using Trizol Reagent (Invitrogen, Carlsbad, CA, USA) according to the manufacturer’s instructions. cDNA synthesis was conducted according to the RNA PCR kit protocol (Invitrogen, Carlsbad, CA, USA). The primer sequences are shown in Table 1. The reaction conditions were 4 min at 95°C, followed by 40 cycles of 94°C for 15 s and 60°C for 25 min. During each extension step, SYBR green fluorescence was monitored and provided the real-time quantitative measurements of the fluorescence. Quantitation was carried out using an external standard curve. Expression levels were calculated by the comparative C_T_ method with glyceraldehyde-3-phosphate dehydrogenase (GAPDH) as an endogenous reference gene.

**Table 1:** Primers used for real-time RT-PCR analysis.

Gene	Sense	Antisense
Collagen I	AGGGACACAGAGGTTTCAGTGGTT	GCAGCACCAGTAGCACCATCATTT
Collagen III	TATCGAACACGCAAGGCTGTGAGA	GGCCAACGTCCACACCAAATTCTT
CTGF	AGACCTGTGCCTGCCATTA	TGTCTCCGTACATCTTCCTG
HGF	TGCTCCCAAATTCCAAAC	GCCATTCCCACGATAACAA
FN	AATGCGTTGGTTTGTACTTGTTATG	CTTCAGCTTCAGGTTTACTCTC
MMP-1	TTTGCCGACAGAGATGAAG	AGCCAAAGGAGCTGTAGATG
TIMP-1	CTGTTCCCACTCCCATCTTT	CTGCTGGGTGGTAACTCTT
SMAD2	TGTCGTCCATCTTGCCATT	CCATCCCAGCAGTCTCTT
SMAD7	CTGCTGTGCAAAGTGTTC	CAGAGTCGGCTAAGGTGAT
α-SMA	TTGAGAAGAGTTACGAGTTG	GGACATTGTTAGCATAGAGG
Lysine hydroxylases	GCTGTTGACTTCCCATTGCT	TCTGATCCAGGTGTCTTTACCC
Prolyo hydroxylase	ACAGGCGGATTGGAAGAGCG	CCCATCCCAAAGCAGTCATCC
GAPDH	CGACCACTTTGTCAAGCTCA	AGGGGTCTACATGGCAACTG

### Statistical Analysis

The data were expressed as the mean ± SEM. Statistical significance was determined with the student’s t-test when there were two experimental groups. For more than two groups, statistical evaluation of the data was performed using one-way analysis of variance (ANOVA), followed by Dunnett’s post-hoc test, with values of *P*<0.05 considered significant.
